# Significance of cadmium from artists’ paints to agricultural soil and the food chain

**DOI:** 10.1186/s12302-016-0077-6

**Published:** 2016-04-21

**Authors:** Nicole Bandow, Franz-Georg Simon

**Affiliations:** BAM Bundesanstalt für Materialforschung und –prüfung, Unter den Eichen 87, 12205 Berlin, Germany

**Keywords:** REACH regulation, Sewage sludge, Fertilizer, Leaching tests

## Abstract

**Background:**

An Annex XV restriction dossier for cadmium in artists’ paints was submitted by an EU member state to the European Chemicals Agency ECHA. By cleaning, used brushes under the tap cadmium can enter the food chain via waste water treatment and subsequent agricultural application of the sewage sludge. It was estimated that 110 kg Cd per year is spread on agricultural land via this exposure route. Other sources of Cd amount to almost 120 tons per year.

**Results:**

The mobility of Cd from pigments was studied in a field-like scenario by leaching experiments using soil samples amended with sewage sludge and spiked with Cd pigments in percolation columns. The redox conditions were confirmed to be the decisive factor for the release of Cd. The release of Cd from artists’ paints was in most cases 1 % or lower in the experiments performed.

**Conclusions:**

Application of sewage sludge containing Cd from artist paints does not increase the amount of Cd leached from this soils. Furthermore, the quantity of Cd from artists’ paints calculated in the restriction dossier is negligible compared to other sources of Cd to agricultural soil. Therefore, ECHA did not consider the proposed restriction to be the most appropriate EU wide measure to address the negligible level of risk.

**Electronic supplementary material:**

The online version of this article (doi:10.1186/s12302-016-0077-6) contains supplementary material, which is available to authorized users.

## Background

Cadmium (Cd) in soils originates from natural and anthropogenic sources. Natural sources comprise volcanic action [1], forest fires, and weathering of Cd-containing rock [2]. There are various anthropogenic sources of Cd: mining and production of non-ferrous metals, production of Cd-containing materials, iron and steel production, coal combustion, waste incineration, and application of mineral fertilizers and sewage sludge in agriculture [3]. The concentration of Cd in agricultural soils is substantially higher than those in non-agricultural soils (with exception of industrial sites with metallurgical industry [4] or mining sites [5]) as a result of the application of Cd-containing phosphate fertilizers [6]. Concentrations in agricultural soils in Europe vary between 0.06 and 0.6 mg/kg [7]. Similar values are observed in the United States (0.1–1.0 mg/kg) [8]. From agricultural soils, Cd is transferred to plants which are known to accumulate Cd from soils and to animals as a result of feeding with Cd-containing food. Cd content of foodstuff varies from 0.01 mg/kg in fruit to 0.5 mg/kg in kidney of cattle, poultry, and pig [9]. The human dietary exposure to Cd can be estimated from the Cd content of respective food items and the intake and has a typical value of 1.5 µg per kg body weight and week (1.8 µg for vegetarians) [10]. Smoking one cigarette package increases the Cd input by 2–4 µg per day [8]. Cd is primarily toxic to the kidney, where it accumulates over time and also causes bone demineralization leading to an increased risk of bone fractures. The hypothesis that dietary exposure to Cd increases the risk of breast cancer is disputed [11].

The European Food safety Authority EFSA considers a tolerable weekly intake (TWI) for Cd of 2.5 µg/kg body weight as appropriate. This is lower by more than a factor of two than the value established by the Joint FAO/WHO Expert Committee on Food Additives (JECFA) with 5.8 µg/kg body weight as weekly intake [12, 13]. Regardless which value is considered the margin between weekly intake of Cd from food and the tolerable intake might be small for certain subgroups of the population, e.g., children or vegetarians. This was the reason for the Swedish Chemicals Agency KEMI to submit a proposal for a restriction according Annex XV of the registration, evaluation, authorisation and restriction of chemicals (REACH) regulation [14] to the European Chemicals Agency ECHA in December 2013. It was proposed to restrict the placing on the market as well as the usage of Cd and its compounds in manufacture of artists’ paints and pigments.

This paper is aimed to investigate whether the presence of Cd pigments could lead to elevated release of Cd to soils. This question is connected to a restriction proposal submitted to ECHA.

### The restriction process under REACH

**Fig. 1:**
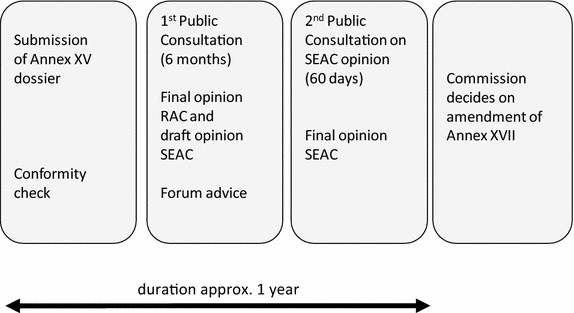
The restrictions process under REACH (simplified)

A restriction of a chemical under REACH can be proposed by the European Commission or by member states if there is an unacceptable risk to human health or the environment resulting from use, manufacture or placing on the market which needs to be addressed on a community-wide basis [14]. Restricted chemicals are listed in Annex XVII of the REACH regulation (Annex XVII, Restrictions on the manufacture, placing on the market and use of certain dangerous substances, preparations and articles). The restriction process under REACH is described in articles 68–73 and is sketched in Fig. 1. To be considered by the European Agency ECHA, the proposal has to meet the regulatory standards of Annex XV (conformity check). Then, the proposal is handled by two separate committees within ECHA. The Committee for Risk Assessment (RAC) summarizes the risk to human health and the environment of the chemical in question and makes a recommendation as to whether the proposed restriction is effective and necessary. The Committee for Socio-economic Analysis (SEAC) evaluates the economic and societal impact of the proposed restriction and the availability of alternatives. The Forum for Exchange of Information on Enforcement examines the restriction proposals with a view to advising on enforceability.

Twelve restriction proposals were submitted to ECHA from 2007 when REACH entered into force up to end of 2013, see Table 1. Until the submission of the restriction proposal on Cd in artists’ paints, only one proposal (Phthalates) was considered not to be justified because the available data do not indicate that there was a risk [15]. When a restriction proposal is supported by the opinions of the two scientific committees RAC and SEAC, the commission decides on a commission regulation, e.g., [16] for the restriction proposal on lead in consumer articles.

**Table 1:** List of proposed restrictions according Annex XV REACH regulation [13] (sorted by date of submission to ECHA)

Date	Substance
15.04.2010	Dimethylfumarate
15.04.2010	Lead and its compounds (in jewellery articles)
15.06.2010	Phenylmercury compounds
15.06.2010	Mercury (in measuring devices)
14.04.2011	Phthalates (DEHP, BBP, DBP, DIHP)
20.01.2012	Chromium (VI) in leather articles
19.04.2012	1,4-Dichlorobenzene
18.01.2013	Lead in consumer articles (which can be placed in the mouth by children)
29.07.2013	Nonylphenol
09.08.2013	1-Methyl-2-pyrrolidone
17.10.2013	Cd in paints (amendment to an existing restriction)
17.01.2014	Cd in artist paints

For Cd and its compounds, various restrictions already exist in Annex XVII of REACH (entry 23, e.g., Cd in plastics); however, there is no entry for Cd in artists’ paints covered by TARIC code (Tarif Intégré de la Communauté) 3213. The pigments used for artists’ paints are PY 35, PO 20, and PR 108 (pigment yellow, pigment orange, and pigment red) and consist of cadmium zinc sulfide Cd_x_Zn_(1−x)_S (PY 35) and cadmium seleno sulfide CdS_(1−x)_Se_(x)_ (PO 20 and PR 108). According to information of CEPE, the European association of paint, printing ink and artists’ colors companies, 39 tons artists’ paints with Cd pigment are sold annually in Europe. The average content of Cd pigments in artists’ paints ranges from 12.1 % in acrylics and some 35 % in oil and water-based paints [17]. Acrylics have the highest market share in terms of quantity with 79 %, followed by oil (14 %), water paints, and gouache (7 %). The Cd quantity in artists’ paints sold in Europe was specified with 6357 kg per annum [17].

### The exposure route

**Fig. 2:**
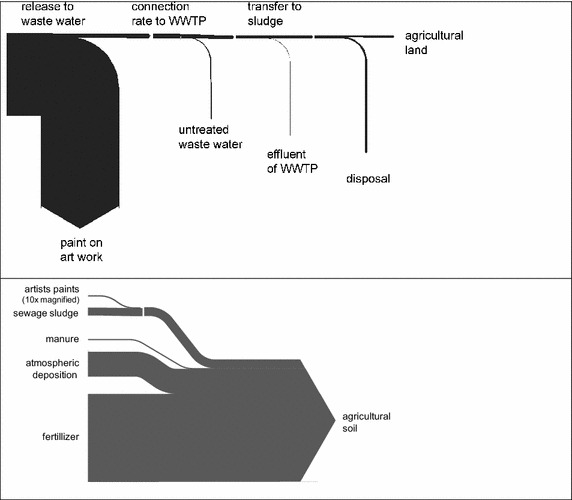
Sankey diagram from the exposure route of Cd originating from artists’ paints to agricultural soil (*top*) and sources of Cd input to agricultural soil (*bottom*). Note: the input flow of Cd from artists’ paint is magnified by a factor of 10 to become better visible. See text for the numeric values of the flows

All numerical data on the exposure route and other sources of Cd to soil are based on the restriction dossier submitted to ECHA [17]. Elevated levels of Cd were observed by the Stockholm Water Association in sewage line outside art schools, most probably as a result of cleaning used paint brushes with residual artists’ paints under the tap. For the exposure estimation [17], the submitter of the restriction dossier assumed a general release factor of 5 %, i.e., 320 kg Cd of the 6375 kg sold would be released in Europe during use of artists’ paints to waste water. The release factor, i.e., to which extent artists’ paints are transferred to waste water, is an estimate of the dossier submitter. No measurements of Cd transfer have been performed. Therefore, the release factor could be an underestimation as well as an overestimation of the real situation. The dossier further assumed that 95 % of the pigments present in the waste water will end up in the sewage sludge in the waste water treatment plants (WWTPs) due to low water solubility of CdS and the acrylic paints itself. Connection rate to WWTPs in Europe was assumed to be 82 %. In average, 45 % of sewage sludge in Europe is used as fertilizer in agriculture. As a result, the dossier submitter concluded that some 110 kg Cd originating from artists’ paints is transferred annually to agricultural soils in Europe (6357 kg × 5 % × 95 % × 82 % × 45 % = 111.4 kg). The described exposure route is displayed in Fig. 2. There are other sources of Cd in urban waste water and thus in sewage sludge accounting to a total of 7.4 tons annually. The removal efficiency of Cd in WWTPs is 60–80 % [18, 19]. However, it can be assumed that Cd from pigments as solid particles are completely removed. Most Cd input to agricultural land arises from mineral fertilizers (85 t/a), followed by atmospheric deposition (24 t/a) and manure (1–2 t/a), so that Cd originating from artists’ paints has a share of 0.09 % on the total Cd input to agricultural soil, see Fig. 2.

During public consultation regarding the restriction dossier (see Fig. 1), more than 600 comments were received by ECHA, much more than in all former restriction processes (see Table 1). Numerous comments were received from professional and hobby artists stating that they do not clean their brushes under the tap rather cleaning rags are used.

## Results

**Fig. 3:**
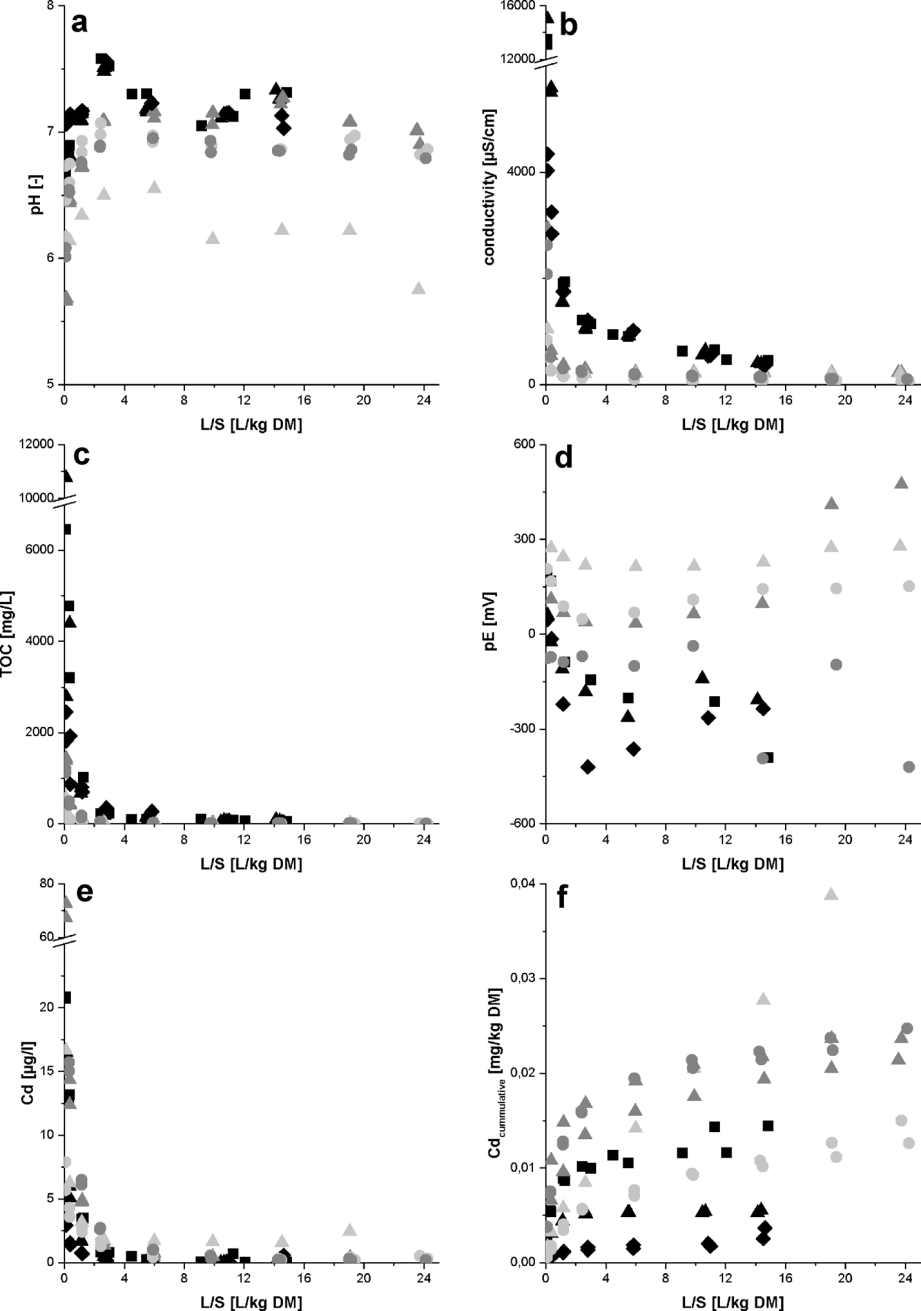
Release in dependence of the liquid-to-solid ratio (L/S) of soils spiked with CdS containing pigments: **a** pH **b** conductivity **c** total organic carbon **d** redox potential (pE) **e** Cd concentration **f** cumulative release of Cd. Different test samples CdSoSlBw10 (*dark squares*), CdSaSl10 (*dark diamonds*) CdSoSl10 (*dark triangles*), CdSoSl1(*grey triangles*), CdSo (*bright triangles*), SoSl1 (*grey circles*) and So (*bright circles*) were used in duplicates

Important characteristics of the test materials are summarized in Table 2. All results of the leaching experiments are shown in Fig. 3. The liquid-to-solid ratio (L/S) refers to the mass of soil filled into the columns for the leaching experiments and to the volume of the percolated leachant, here artificial rainwater. The pH value of all eluate samples was neutral or slightly acidic with a minimum pH of 5.7. In the course of all column tests, the pH value of the eluates increases somewhat up to an L/S of about 5 L/kg and decreases then only slightly in the further fractions. In general, the pH value of the eluate fractions increased with the amount of sewage sludge present in the sample. This indicates that addition of sludge increases the buffering capacity of the soil sample. The conductivity is dependent of the content of sludge and biowaste in the sample. Values in the first fraction differ by one order of magnitude: 3300 µS/cm (CdSoSl10Bw10), 12,700 µS/cm (CdSoSl10), 4200 µS/cm (CdSaSl10); 2900 µS/cm (CdSoSl1), 2000 µS/cm (SoSl1), and 1000 µS/cm (CdSo), respectively. All samples show fast declining conductivity around 1000 µS/cm at a L/S of 2.6 L/kg for samples with 10 % sludge and between 120 and 220 µS/cm for samples with 1 % sludge or less. The two following fractions also reached constant conductivities at the end of the experiment. The TOC shows a similar concentration profile as the conductivity. The TOC concentration decreases fast with higher L/S ratios and is also depended on the carbon content of the samples. The concentration in the first fraction ranges from 6800 mg/L (CdSoSl10Bw and CdSoSl10) to 260–560 mg/L (CdSo and So). In all samples with 1 % sludge or less, the TOC reaches concentrations less than 10 mg/L, while the concentration in the other samples is between 40 and 90 mg/L. The redox potential in the eluates depends on the amount of sludge added to the sample and is in general lower with increasing amount of organic carbon. All samples with 10 % sludge start with a positive redox potentials between 36 and 195 mV and show a fast decrease to negative potentials in the second (CdSoSl10 and CdSaSl10) or third fraction (CdSoSl10BW10), while soil samples without amendment stay in the positive range during the whole experiment. Samples containing 1 % sludge show higher variance without a clear trend. Cd concentrations in all eluates are in the µg/L range starting between 7 and 70 µg/L. The decrease to lower concentrations is more pronounced for samples with higher sludge content and the CdSo sample is the only one showing concentrations above 0.6 µg/L in the last fraction. Due to the different initial Cd concentrations in the soil samples, the cumulative release of Cd is more meaningful: The cumulative release is small (0.003–0.059 mg/kg) in comparison to the total content of Cd (1.3–2.9 mg/kg) and at the end of the leaching experiments less than 2 % of the present Cd was released (Table 3). The calculated standard deviation for the release is 10 % or lower. An exception is the sample CdSaSl10 showing a deviation of 20 %. Samples containing 10 % sludge release less than 0.3 % of total Cd present, while the CdSoSl10Bw10 sample, containing biowaste additionally, released 0.66 %. The soil sample itself releases about 1 % of the present Cd, this percentage is slightly increased to 1.35 % after spiking of the soil sample. In contrast to this, the highest percentage was released from the SoSl1 sample, which is not further increased by spiking with Cd.

**Table 2:** Overview about composition of materials used for column percolation test and properties of these materials

Sample	Cd-spiked	Sewage sludge [%, DM]	Biowaste [%, DM]	Sand [%]	Soil [%]	Cd content [mg/kg]	Loss on ignition [%]
CdSoSl10Bw10	Yes	10	10	–	80	1.96	9.98
CdSoSl10	Yes	10	–	–	90	1.84	7.67
CdSoSl1	Yes	1	–	–	99	2.26	2.58
SoSl1	No	1	–	–	99	1.32	2.56
CdSo	Yes	–	–	–	100	2.87	2.07
So	No	–	–	–	100	1.33	2.07
CdSaSl10	Yes	10	–	90	–	1.40	5.76

Numbers behind abbreviations indicate amount [%] added to the soil

*DM* dry matter; *Cd* sample is spiked with Cd-containing pigments; *So* soil; *Sl* sludge; *Bw* biowaste; *Sa* sand

**Table 3:** Total Cd content in soil material, cumulative release of Cd of these soil materials during column percolation test and release in [%] of total content

Sample	Cd content [mg/kg]	Cumulative release [mg/kg]	[%]
CdSoSl10Bw10	1.96	0.013	0.66
CdSoSl10	1.84	0.005	0.29
CdSoSl1	2.26	0.023	1.02
SoSl1	1.32	0.024	1.81
CdSo	2.87	0.057	1.98
So	1.33	0.014	1.05
CdSaSl10	1.40	0.003	0.21

## Discussion

In our investigation, Cd originated from three sources: (1) Cd present in the soil sample itself, (2) Cd in the sewage sludge added, and (3) from spiking. Only a small portion of the Cd is released during the leaching experiments, from which source could not be revealed. The comparison between release from spiked and non-spiked samples does not show elevated release from the spiked samples. As discussed earlier, two main mechanisms are possible for the release: dissolution of CdS by acids or oxidizing of the sulfide to sulfate. All eluates are not acidic enough for dissolving CdS (pH = 3.5, see Fig. 5). It seems rather unlikely that the pH value of seepage water in typical agricultural soils reaches pH values low enough to dissolve CdS taking into consideration that the artificial rain water used in this experiment has a higher acidity (pH approx. 3) than natural rain water to simulate a worst case situation and that the sandy soil sample has quite a small buffering capacity for acids. In contrast to this, the observed redox potential is above −150 mV in all samples besides CdCoSl10, CdSoSl10Bw10, and CdSoSl10, and thus oxidizing enough to dissolve CdS. This is reflected by the lower release percentage (0.21–0.66 %) in these three samples in contrast to the other samples (>1 %). The redox conditions are obviously more decisive in our investigations for the release of Cd.

It is obvious that Cd from artists’ paints displays a very small part of the total Cd input to agricultural soil. The future trend of Cd concentrations in European soils is expected to be negative in average (−15 % over the next 100 years) [20]. Reasons for that are reduced use of fertilizers and less Cd input from atmospheric deposition as a result of effective emission control in combustion and incineration plants. The potential and cost-effectiveness of measures to reduce discharges and emissions of Cd and other hazardous substances were studied by the Baltic Marine Environment Protection Commission. The improvement of air abatement measures has the highest reduction potential in small and medium thermal plants (<50 MW). These emissions could be cut by 50 % at costs of 50–70.000 EUR per kg Cd [21] and will lead to less atmospheric deposition of Cd.

A reduction of the Cd content in urban waste water und thus in sewage sludge is difficult to obtain because it originates from various single sources. To a large extent, these are unknown. Sörme et al. estimated the sources of Cd in a Stockholm waste water plant [22]. According to that 30 % were from car washes, 10 % from artists’ paints, 9 % from food, and 39 % unknown. Measurements in Scandinavian countries of Cd arising from car washes were published [23]; however, it remains unclear which part of a vehicle emits Cd in a car washing plant. One possible source from cars could be parts of the brake system [24]. In general, today’s sources of heavy metals in waste water from household are poorly understood [25]. Decades ago, the Cd content in sewage sludge was considerably higher, e.g., in Paris by a factor of 27 [26].

In the restriction dossier [17], a value of 1.4 mg Cd/kg sewage sludge (dry matter, median of 18 EU countries ranging from <0.4 to 4) applied to agricultural soils was used for all calculations. The value for Cd in sewage in Germany was 1.0 mg/kg which fits well to the sewage sludge survey of the German Association for Water, Wastewater and Waste (DWA) from 2003 (median Cd content 1.15 mg/kg) [27]. The median value for the German Federal state Mecklenburg-Vorpommern is even lower (0.65 mg/kg) [28]. According to the German Ordinance on Fertilizers [29] a limit value of 1.5 mg Cd/kg was set into force from January 1st, 2015 for sewage sludge in agricultural applications. This limit value excludes some 5 % of the sludge generated in Mecklenburg-Vorpommern (97,000 ton per year) from direct application in agriculture [28]. However, due to the new limits in the Fertilizer Ordinance, some 20 % of the sewage sludge generated are excluded now from agricultural application because of a mercury content above 1 mg/kg, also. Exclusion of highly contaminated sewage sludge by setting stringent limit values seems to be an effective measure to reduce contaminant transfer to agricultural soil at least for heavy metal contamination. However, there are also organic pollutants in sewage sludge such as emerging pollutants of concern (EMPOC), pharmaceuticals and personal care products (PPCP), among others antibiotics, endocrine disruptors, fragrances, UV filters, and antiseptics which are released to the environment [30] and where no limits exist so far. That is the reason that agricultural application of sewage sludge is under discussion in several EU countries. In Germany, Belgium, Slovenia, and The Netherlands, the majority of the sludge (55–70 %) is incinerated (Eurostat data from 2013, database env_ww_spd). But whereas organic pollutants are destroyed during incineration, heavy metals remain in the ash. According to a survey on sewage sludge ash in Germany, the resulting ash contains 1.4 mg Cd/kg (median, values from <0.1 to 80.3 mg/kg) [30]. Processes to enable recovery of phosphorus from sewage sludge ash for fertilizer production after removal of heavy metals are available [31].

**Fig. 4:**
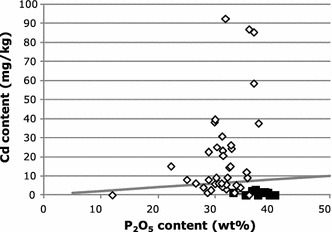
Cd concentration in phosphate rock in relation to phosphorus content (data from [50]). The concentrations in igneous rock (*filled squares*) are considerably lower than in sedimentary phosphate rock. Straight line at 20 mg Cd/kg P_2_O_5_ indicates a possible limit value for Cd in fertilizers

The largest contribution of Cd input to agricultural soils originates from mineral fertilizers. Phosphate rock as raw material for fertilizer production often contains Cd and other heavy metals like Uranium which form sparingly soluble phosphates. Sedimentary phosphate rock contains considerably more Cd than igneous rock, see Fig. 4. A similar figure is shown for fertilizers in the work from Nziguheba and Smolders [32]. Setting a limit for Cd in fertilizers would be a possible measure to reduce the Cd input to agricultural soils, e.g., 20 mg/kg P_2_O_5_ (see straight line in Fig. 4). All igneous raw materials fulfill this limit, but the majority of the sedimentary phosphate rock bears already above 20 mg Cd/kg P_2_O_5_. Instead of a limit which excludes materials from application, a tax on the Cd content above the limit is conceivable. This approach was applied from 1995 to 2010 in Sweden and is discussed in detail elsewhere [33]. This measure might have been successful, whereas the European average of the Cd input to arable soils via phosphate fertilizers was 1.6 g/ha/y, in Sweden this value was as low as 0.1 g/ha/y [32].

## Conclusions

The addition of Cd pigment to the samples in the present investigation does not lead to an elevated release. However, concerning the long-term behavior, it might be possible that Cd from artists’ paints is mobilized either by oxidation of sulfide to sulfate or by dissolution at lower pH values. The mobilized Cd (Cd^2+^) will then be transferred via the soil–plant pathway (intake by crops) or via the soil–water pathway leaching, i.e., the outflow of Cd from the top soil. The underlying processes are described in detail elsewhere [34]. In this paper, the behavior of Cd in soil was investigated. The quantity of Cd arising from artists’ paints calculated in the restriction dossier, amounting to 110 kg per year [17], is negligible compared to other sources of Cd to agricultural soils (almost 120 tons/a).

That was the reason that RAC and SEAC, the scientific committees at ECHA did not consider the proposed restriction to be the most appropriate EU wide measure to address the negligible level of risk in terms of its effectiveness in reducing the risks from cadmium in artists’ paints [17]. Meanwhile, the European commission has terminated the restriction process on Cd in artists’ paints [35].

Chemicals are released to the environment during production, use, and waste management of products. This release has to be restricted by regulations and limit values securing human health and minimizing negative impacts on the environment. In spite of this effort, it is also clear that zero emissions are not possible and thus it is necessary to focus on the main exposure routes to achieve a better protection. The example of CdS containing paint pigments clearly shows that it is necessary to include both natural sciences and socioeconomic aspects for a meaningful decision. It also demonstrates the importance of including the consumers into the risk assessment as in this case release to the waste water can easily be avoided, in this case by not cleaning brushes with artists’ paints under the tap.

## Methods

All used chemicals were of p.a. (pro analysi) quality or higher. Details are given in the Additional file 1. Artist’s paints containing Cd pigments for spiking were bought at a local craft store (cadmium yellow light, pigment PY35, Lukas Cryl pastos, Düsseldorf, Germany).

Soil samples were taken on an agricultural used field in Güterfelde near Berlin, Germany. The samples were air-dried and sieved to a particle size < 2 mm after manual removal of stones and roots. For specification of soil type, the grain size distribution was determined [36]. The soil carbonate content was assessed according to the following standards [37]. Details on the methods are given in the Additional file 1. According to grain size distribution, soil type can be classified as a sandy soil with 96 % of grains between 0.063 and 1 mm (matching a medium sand with high amount of fine sand). The carbonate content of the soil is 0.35 %. The organic carbon content of the soil is relatively low with 2 % and was increased up to 10 % by adding sludge and biowaste.

The soil was mixed with different amounts of sewage sludge and biowaste to vary the carbon content and the resulting redox conditions in the sample (see Table 2). The sewage sludge originates from the WWTP Wassmannsdorf (near Berlin, Germany) and was sampled in November 2011. The biowaste was obtained from a commercial company and presents compost produced from fermentation of plant garbage in a treatment plant in Saxony-Anhalt, Germany. Both materials were air-dried and sieved to a particle size <2 mm. The Cd pigments were suspended in water and 100 g of sewage sludge and 200 g soil, respectively, was added while stirring the suspension. While this mixture was air-dried, it was mixed regularly until the solid was dry enough for further processing. The spiked sludge and soil were mixed with soil, sludge, and biowaste to achieve mixtures according to Table 2. All mixtures were homogenized using a gyro wheel mixer (Engelsmann, Ludwigshafen, Germany) for 2 h and subdivided using an automated sample divider (Retsch, Haan, Germany).

### Characterization of test materials

The Cd contents in the paint, soil, biowaste, sewage sludge, and all Cd-spiked samples were determined after digestion with aqua regia using an ICP-OES (iCap 7400 Duo, ThermoScientific, Dreieich, Germany) with matrix-matched external calibration. For characterization of the sample materials, the water content [38], the bulk density [39], and loss of ignition [40] were also determined. Further details on analytical methods are presented in the Additional file 1.

### Geochemical behavior of cadmium sulfide

**Fig. 5:**
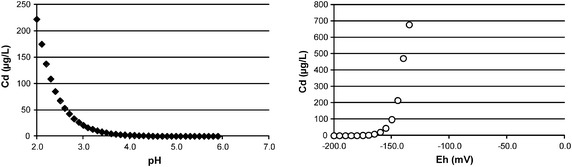
Solubility of 10^−5^ mol/L CdS as function of pH (*left*) and redox potential *E*_h_ (*right*)

Cd belongs to the hydrogen sulfide group, thus CdS forms already at acidic pH values. It has a very low solubility product (K_s_ = [Cd^2+^][S^2−^]=1.0 × 10^−28^ mol^2^/L^2^) [41]. However, solubility of Cd increases with decreasing pH value. Further, sulfides can be oxidized under natural conditions by electron acceptors such as O_2_ or Fe^3+^. This behavior can be described by geochemical modeling with the program MinteqA2 [42]. The solubility of 10^−5^ mol/L CdS (Greenockite) as function of pH from 2 to 6 and the redox potential *E*_h_ starting from −200 mV is displayed in Fig. 5. It can be seen that Cd concentrations in solution increases with more acidic pH values and under more oxidic conditions.

### Column leaching tests

All column leaching tests were performed in duplicates according to DIN 19528 [43]. Column leaching test show a good reproducibility (deviation between replicates in general <20 %) and robustness [44]. Glass columns were filled with the sample material including filtering layers of sand at the top and bottom of the column. The contact time between sample and eluent was set constant at 5 h. The water saturated columns were percolated with artificial rainwater with a pH of approx. 3 [45]. The pH value was chosen lower than under natural conditions to accelerate the consumption of the acid neutralization capacity of the soil samples. The exact composition of the rain water is given in Additional file 1: Table S2. Eluate fractions were sampled at fixed L/S ratios: 0.1; 0.35; 1.1; 2.6; 5.8; 10; 14; 19 and 24 L/kg. The chosen L/S ratios differ from the ones defined in the standard because the maximum duration until an L/S of 4 L/kg is not long enough to answer our research question. Small variations for the different sample materials were necessary to keep sampling times in normal laboratory working hours. Further details on test conditions can be found in Additional file 1: Table S1.

### Analysis of eluates

Redox potential of eluates was measured online with an EMC 30 electrode (Meinsberg, Waldheim, Germany) at the outflow of the column for one replicate of each material. All fractions were divided into aliquots for the analytical procedures described in the following section. Conductivity [46], pH, turbidity [47], and DOC (DIN EN 1484) were measured immediately after sampling, while the samples for cation analysis were acidified using concentrated HNO_3_ and samples for anion analysis were stored at 4 °C. Concentration of anions was determined using ion chromatography with electrical conductivity detector (DIN EN ISO 10304-1:2009-01), while for cations, ICP-OES [48] or IPC-MS [49] was used. Further details on analytical methods are given in the Additional file 1.

## Additional file

10.1186/s12302-016-0077-6 Details on analytical methods are available online.

## Abbreviations

REACH: registration, evaluation, authorisation and restriction of chemicals
ECHA: European Chemicals Agency
SEAC: socioeconomic analyses committee at ECHA
RAC: risk assessment committee at ECHA
EFSA: European food safety authority
ICP-OES: inductively coupled plasma-optical emission spectrometry
ICP-MS: inductively coupled plasma-mass spectrometry
WWTP: waste water treatment plant
L/S: liquid-to-solid ratio (L/kg)
