# Supplementary material for: Significance of cadmium from artists’ paints to agricultural soil and the food chain
Source: Environ Sci Eur. 2016 Apr 21;28(1):12. doi: 10.1186/s12302-016-0077-6 (PMC5044963; doi:10.1186/s12302-016-0077-6)
Supplement: Supplementary file 1 — 10.1186/s12302-016-0077-6 Details on analytical methods are available online. [file 12302_2016_77_MOESM1_ESM.pdf]

1. Characterization of test material

1.1 Aqua regia digestion and ICP-OES

Microwave containers made of Teflon were used for microwave digestion. Approx. 0.5 of sample were moistened with 2 ml ultrapure laboratory water (Ultra Clear System, Siemens, Berlin, Germany) and 3 ml nitric acid (65%) and 9 ml hydrochloric acid (37%) were added (pro analysis, Merck, Darmstadt, Germany). The following micro wave program was used: The sample was heated in 6 min up to 160 °C and then in 4 °C up to 220 C° using 1000 W and hold for 20 min and 700 W at this temperature. The sample was vented afterwards for 40 min. Samples were filtered after digestion, quantitatively transferred to 50 ml volumetric and filled with ultrapure water. All samples were diluted by a factor of 10 before analysis with ICP-OES. A matrix matched calibration with five calibration levels was used for quantification. Calibration solutions were prepared using Certipur® single element standards (Merck).The sample was introduced in the system using a nebulizer with an argon (5.0) gas flow rate of 0.5 ml/min and auxiliary gas flow rate of 1.5 L/min. For quantification the signal at a wavelength of 228.802 nm with axial direction of plasma view was and an exposure time of 15 s (UV) and 5 s (VIS) was used.

1.2 Grain size distribution,Water content, loss of ignition and bulk density, carbonate content and organic carbon content

Approx. 200 g of air dried soil sample were sieved using a set of sieves including 4 mm, 2 mm, 0.63 mm, 0.2 mm, 0.125 mm and 0.063 mm to obtain the grain size distribution. The result is shown in Fig.S1. The soil can be characterized as well-graded sand consisting of mainly medium sand and a high amount of fine sand. For determination of water content, 5 g of sample was used and heated at 105 °C until constant mass was achieved using a MA30 (Sartorius, Göttingen, Germany) moisture measuring device. 20 g of sample, which were dried at 105 °, were heated for 3 h at 550 °C using a muffle furnace for determination of loss of ignition. For bulk density measurement, 5 g of dried and milled sample were placed in the measurement chamber of a helium pycnometer AccuPyc 1330 (Micrometrics, Aachen, Germany). Volume of the sample was determined by displacement of Helium by the sample. All measurements were performed in duplicates. The carbonate content was analyzed following the principle of DIN ISO 10693:2014-06. Therein the carbonate content of the sample is determined by the amount of CO_2, which is produced after reaction with hydrochloric acid. 10 g of air dried soil were exposed to 10 ml of 10 % hydrochloric acid (technical grade) and the produced gas is measured with a sensor. In comparison to the respective standard an improved carbonate measuring device was used (company D. Klosa, Hambühren, Germany) equipped with a gas pressure sensor as well as automated temperature and atmospheric pressure adjustment. For determination of the carbon content a Vario MACRO CHN (Elementar Analysensysteme GmbH, Hanau Germany) elemental analysator was used. Approx. 100 mg of the air dried sample is catalytically combusted in an oxygen atmosphere. After removal of other gases as e.g. SO_2, specific adsorber columns are used for separation of the analytes. All analytes are detected using a thermal conductivity detector.

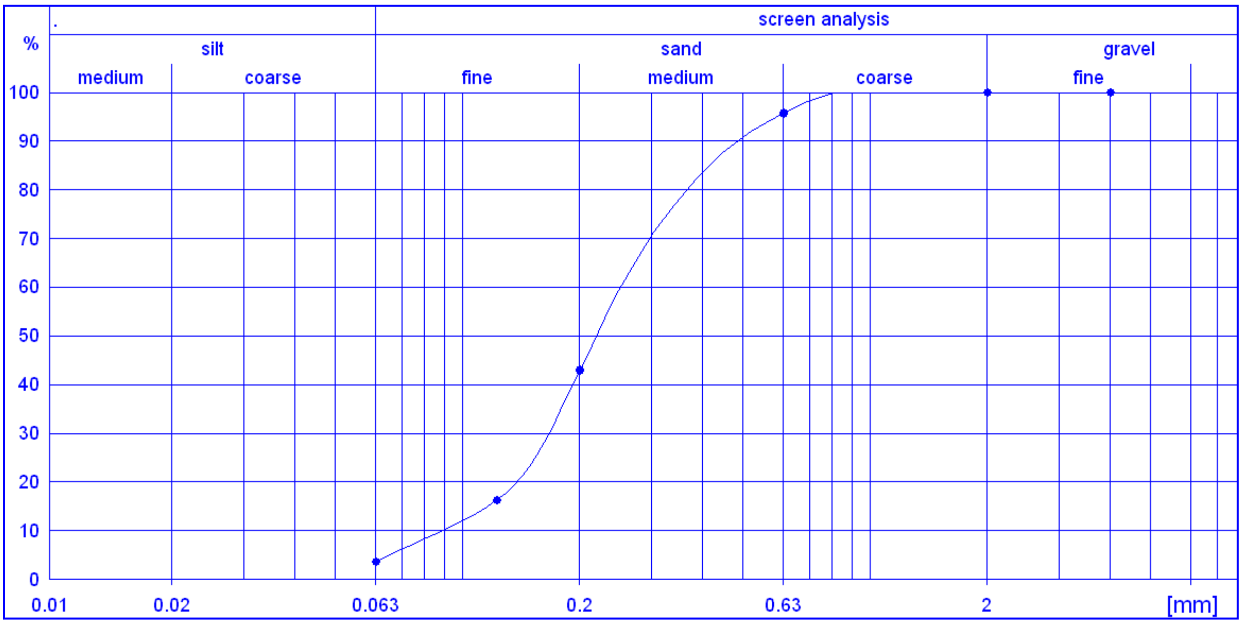

Screening of sample

Grain size [mm]	Mass of Residuals [g]	Sieve residue [Wt.-%]	Sum of Screen underflow [Wt.-%]
4	-	-	100,00
2	0,05	0,02	99,98
0,63	8,36	4,11	95,87
0,2	107,43	52,79	43,07
0,125	54,49	26,78	16,30
0,063	25,96	12,76	3,54
< 0,063	7,2	3,54	-

Tot. dry mass: 203,49 g
Sum: 203,49 g
Loss of screening:
Type of screening: Trocken

Figure S 1: Grain size distribution of soil used in the percolation tests.

2. Percolation Tests

A contact time between sample and eluent of 5 h is specified for column leaching experiments according to DIN 19528:2009-01. The flow rate has to be calculated as shown in equation S1. Due to different bulk densities of the different test materials and differences in column packing the flow rate has to be varied to achieve constant contact times (Table S1).

$$q = \frac{v * n}{t_c * 60} \quad \textbf{equation S 1}$$

with q: flow rate [ml/min]; V: Volume filled with sample material [ml]; n: porosity [-]; t_c: contact time [h]; 60: time conversion factor

The porosity is calculated using the bulk density (p_0, g/cm^3) of the sample material and the particle density (p_d, g/cm^3) of the packing in the column:

$$n = 1 - \frac{p_d}{p_0} \quad \textbf{equation S 2}$$

Table S 1: Column dimensions, sample filling, flow rate and resulting contact time during column percolation tests.

Sample	Inner diameter column [cm]	Sample dry mass [g]	Filling height sample [cm]	Bulk density sample [g/cm³]	Porosity [%]	Flow rate [ml/min]	Contact time sample/ eluent [h]
CdSaSl10BW10	5.86	955	26.5	2.41	0.45	1.08	5
CdSaSl10	5.86	1223	29.5	2.46	0.38	1.00	5
CdSo	5.86	1153	27.5	2.61	0.40	0.97	5
CdSoSl1	5.86	1153	27.5	2.61	0.40	0.97	5
So	5.86	1153	27.5	2.61	0.40	0.96	5
SoSl	5.86	1153	27.5	2.61	0.40	0.95	5

In all experiments artificial rainwater (see table S2) was used as leachant. For preparation of the rainwater the following chemicals were used: KCl, $CaSO_4*2H_2O$, NH_4Cl, $NaNO_3$, $MgSO_4*7H_2O$, and HNO_3 (all pro analysi, Merck).

Table S 2: Chemical composition of artificial rain water used in column percolation tests.

ion	concentration [mg/L]
Na^+	1.38
NH_4^+	0.72
K^+	1.56
Ca^{2+}	2.00
Mg^{2+}	0.24
Cl^-	2.84
NO_3^{2-}	127
SO_4^{2-}	5.76

3. Analysis of eluates

3.1 pH, conductivity and turbidity

Conductivity was measured using a TetraCON 96 electrode and pH using a SenTix 41 electrode (both WTW, Weilheim, Germany). For determination of turbidity a 2100 AN IS turbidimeter from Hach (Düsseldorf, Germany) was applied.

3.2 DOC

Eluates samples were filtered using a 0.45 µm filter and the DOC concentration was determined according to DIN EN 1484 (DIN EN 1484, 1997-08) using a TOC VCPH (Shimadzu, Duisburg, Germany) by subtraction of the dissolved inorganic carbon (DIC) from the dissolved carbon (DC):

DOC=DC-DIC   equation S3

3.3 Cations

Cd concentrations in the eluates were measured using an ICP-OES with external calibration. Calibration solutions were prepared using Certipur® single element standards (Merck, Germany). The sample is introduced to the system using a nebulizer with an argon (5.0) gas flow rate of 0.5 ml/min and auxiliary gas flow rate of 1.5 L/min. The signal at a wavelength of 228.802 nm with axial direction of plasma view and an exposure time of 15 s (UV) and 5 s (VIS) was used for quantification. If concentrations were below the detection limit, these fractions were also analyzed using an ICP-MS with external calibration. Samples were introduced into the system using an ESI SC-2DX auto sampler with Fastventil. Cool gas flow was set to 14 L/min, auxiliary flow to 0.8 L/min and the nebulizer flow to 1.02 L/min. ^{115}Indium was used as internal standard and all samples were measured using the KED-modus with 60 sweeps. ^{110}Cd was used for quantification.
